# Supplementary material for: Changes in taxonomic and phylogenetic diversity in the Anthropocene
Source: Proc Biol Sci. 2020 Jun 17;287(1929):20200777. doi: 10.1098/rspb.2020.0777 (PMC7329034; doi:10.1098/rspb.2020.0777)
Supplement: Supporting figures and tables [file rspb20200777supp1.pdf]

Supplementary of: Changes in Taxonomic and Phylogenetic Diversity in the Anthropocene

Daijiang Li[1*], Julian D. Olden[2], Julie L. Lockwood[3], Sydne Record[4], Michael L. McKinney[5], and Benjamin Baiser[1]

18 May, 2020

Journal: Proceedings B
DOI: 10.1098/rspb.2020.0777

This supplementary information file includes:

```
Phylogeny building process
Table S1 to S5
Figs. S1 to S9
Text S1
```

Phylogenies

For plants, we generated the phylogeny using Phylomatic v4.2 [1] based on the synthesis phylogeny `zanne2014` [2], which was constructed and time-calibrated from seven gene regions for >32000 plant species using maximum-likelihood. We chose this phylogeny because it is one of the most up-to-date phylogenies for plants.

For birds, because we had extinct birds in the historical data period [3], we used the 100 augmented phylogenies constructed by Baiser et al [4]. They augmented 100 randomly selected global bird phylogenies generated by Jetz et al [5] by inserting extinct birds into the phylogenies in a position from the stem branch preceding the most recent common ancestor of species from the same family. The branch length of the inserted species extended to the present time to make the augmented phylogenies ultrametric. We analyzed each dataset with all 100 augmented phylogenies and then used the average values as final measurements. Previous studies [4,6] suggested that 100 phylogenies were enough to get stable phylogenetic diversity values.

For fishes, we were unable to find a phylogeny built with gene sequences that was large enough to contain most of the species in our datasets. The most recent large fish phylogeny [7] had 11,638 species. However, only 6,115 out of 13,236 fishes in our datasets were included in this phylogeny. Therefore, we instead extracted a phylogeny for our fishes from the Open Tree of Life [8], which is a comprehensive phylogeny with ~2.3 million tips by synthesizing published phylogenies. However, the phylogeny extracted from the Open Tree of Life did not have branch lengths (i.e., not a chronogram), which is required for most phylogenetic diversity measures. To calculate branch length, for each of the 3,404 internal nodes, we first derived its descendants and then searched for their divergence time through the TimeTree of Life database [9]. The TimeTree database was

compiled based on 3,163 studies and 97,085 species (as of October 10, 2017). For each pair of species included in their database, we extracted their average divergence time from all previous studies. In the end, we extracted divergence time for 767 internal nodes. With the phylogeny from the Open Tree of Life and the divergence date of internal nodes from the TimeTree database, we then solved the branch length using Phylocom [10] and its `bladj` function. The `bladj` algorithm placed estimated node ages onto the phylogeny. Ages of nodes without dates were then estimated by equally placing ages between nodes with dates to minimize variance in branch lengths.

These final phylogenies, however, still did not cover all species in our datasets. This was because there were taxa not identified to species level or species that were not included in the Open Tree of Life database. In the end, we had 31131 out of 32382 (96.14%) plant species, 2399 out of 2903 (95.66%) bird species, and 12448 out of 13236 (94.05%) fish species in their corresponding phylogenies. Therefore, we calculated the proportion of species in the phylogeny for each dataset. We removed datasets that had less than 80% of their species covered in the phylogeny from any 'time period' (`dat_1` and `dat_2`), resulting in 162 out of 189 datasets for final analyses (Supplementary Table S5). For these 162 datasets, we removed the small fraction of species that were not in the phylogeny prior to analyses.

References

1. Webb CO, Donoghue MJ. 2005 Phylomatic: Tree assembly for applied phylogenetics. *Molecular Ecology Notes* **5**, 181–183.

2. Zanne AE *et al.* 2014 Three keys to the radiation of angiosperms into freezing environments. *Nature* **506**, 89–92.

3. Cassey P, Lockwood JL, Blackburn TM, Olden JD. 2007 Spatial scale and evolutionary history determine the degree of taxonomic homogenization across island bird assemblages. *Diversity and Distributions* **13**, 458–466.

4. Baiser B, Valle D, Zelazny Z, Burleigh JG. 2018 Non-random patterns of invasion and extinction reduce phylogenetic diversity in island bird assemblages. *Ecography* **41**, 361–374.

5. Jetz W, Thomas G, Joy J, Hartmann K, Mooers A. 2012 The global diversity of birds in space and time. *Nature* **491**, 444–448.

6. Li D, Monahan WB, Baiser B. 2018 Species richness and phylogenetic diversity of native and non-native species respond differently to area and environmental factors. *Diversity and Distributions* **24**, 853–864.

7. Rabosky DL *et al.* 2018 An inverse latitudinal gradient in speciation rate for marine fishes. *Nature* **559**, 392–395. (doi:10.1038/s41586-018-0273-1)

8. Hinchliff CE *et al.* 2015 Synthesis of phylogeny and taxonomy into a comprehensive tree of life. *Proceedings of the National Academy of Sciences* **112**, 12764–12769.

9. Kumar S, Stecher G, Suleski M, Hedges SB. 2017 TimeTree: A resource for timelines, timetrees, and divergence times. *Molecular Biology and Evolution* **34**, 1812–1819.

10. Webb CO, Ackerly DD, Kembel SW. 2008 Phylocom: Software for the analysis of phylogenetic community structure and trait evolution. *Bioinformatics* **24**, 2098–2100.

Table S1. Summary of LMM output for effect sizes of α and β diversity for different taxonomic groups. Significant changes (i.e., 95% confidence interval does not include zero) are in bold. Abbreviations: SR, species richness; PD, Faith's phylogenetic diversity (without root); PSV: phylogenetic species variation; Sorensen_tur, Turnover component of Sorensen dissimilarity; pSorensen_tur, Turnover component of Phylogenetic Sorensen dissimilarity; PCDp, phylogenetic component of phylogenetic community dissimilarity.

index	Changes in diversity	Estimate	Std. Error	2.5 %	97.5 %	Random Terms
α: Bird (54)						
SR	6.18 %	0.060	0.061	-0.059	0.179	study; grain_size
PD	3.37 %	0.033	0.051	-0.066	0.132	study; grain_size
PSV	-2.08 %	-0.021	0.013	-0.047	0.005	study; grain_size
α: Fish (55)						
**SR**	**10.62 %**	**0.101**	**0.049**	**0.005**	**0.197**	**study**
**PD**	**8.21 %**	**0.079**	**0.037**	**0.007**	**0.151**	**study**
**PSV**	**-0.11 %**	**-0.001**	**0.000**	**-0.002**	**0.000**	**study**
α: Plant (50)						
SR	4.69 %	0.046	0.065	-0.081	0.173	study
PD	2.84 %	0.028	0.101	-0.170	0.226	study; data_type
**PSV**	**-0.77 %**	**-0.008**	**0.003**	**-0.014**	**-0.002**	**study**
β: Bird (54)						
Sorensen_tur	-4.58 %	-0.047	0.092	-0.228	0.134	study; data_type; driver
pSorensen_tur	-5.16 %	-0.053	0.063	-0.177	0.071	study; data_type; driver
PCDp	1 %	0.010	0.014	-0.018	0.038	study
β: Fish (55)						
**Sorensen_tur**	**-8.89 %**	**-0.093**	**0.047**	**-0.185**	**-0.002**	**study**
pSorensen_tur	-2.43 %	-0.025	0.037	-0.098	0.049	study
**PCDp**	**0.6 %**	**0.006**	**0.001**	**0.004**	**0.008**	**study**
β: Plant (50)						
**Sorensen_tur**	**-6.37 %**	**-0.066**	**0.031**	**-0.126**	**-0.005**	**study**
**pSorensen_tur**	**-5.47 %**	**-0.056**	**0.028**	**-0.112**	**-0.001**	**study**
PCDp	-0.44 %	-0.004	0.009	-0.022	0.013	study

Table S2. Summary of LMM output for effect sizes of α and β diversity diversity for different sampling methods. Significant changes (i.e., 95% confidence interval does not include zero) are in bold. Abbreviations: SR, species richness; PD, Faith's phylogenetic diversity (without root); PSV: phylogenetic species variation; Sorensen_tur, Turnover component of Sorensen dissimilarity; pSorensen_tur, Turnover component of Phylogenetic Sorensen dissimilarity; PCDp, phylogenetic component of phylogenetic community dissimilarity.

index	Changes in diversity	Estimate	Std. Error	2.5 %	97.5 %	Random Terms
α: Land Use Data (66); land use gradient approach						
**SR**	**-10.22 %**	**-0.108**	**0.052**	**-0.209**	**-0.007**	**study**
**PD**	**-12.29 %**	**-0.131**	**0.041**	**-0.212**	**-0.050**	**study**
**PSV**	**-3.31 %**	**-0.034**	**0.011**	**-0.055**	**-0.013**	**study**
α: Land Use Data (66); landscape approach						
SR	-1.04 %	-0.011	0.025	-0.060	0.039	study; taxa; driver
PD	-2.35 %	-0.024	0.024	-0.070	0.023	study; taxa; driver
**PSV**	**-1.05 %**	**-0.011**	**0.003**	**-0.017**	**-0.004**	**study; taxa; driver**
α: Resample Data (18)						
SR	17.09 %	0.158	0.093	-0.024	0.339	study
PD	15.18 %	0.141	0.074	-0.003	0.286	study
PSV	1.19 %	0.012	0.012	-0.012	0.036	study
α: Species List Data (78)						
**SR**	**6.93 %**	**0.067**	**0.016**	**0.036**	**0.098**	**study**
**PD**	**4.59 %**	**0.045**	**0.010**	**0.025**	**0.064**	**study**
**PSV**	**-0.25 %**	**-0.002**	**0.001**	**-0.005**	**0.000**	**study**
β: Land Use Data (66); land use gradient approach						
Sorensen_tur	-6.24 %	-0.064	0.095	-0.251	0.122	study; taxa; driver
pSorensen_tur	-5.56 %	-0.057	0.071	-0.197	0.083	study; taxa; driver
PCDp	0.55 %	0.005	0.006	-0.006	0.017	study; taxa; driver
β: Land Use Data (66); landscape approach						
**Sorensen_tur**	**7.88 %**	**0.076**	**0.035**	**0.008**	**0.144**	**study; taxa; driver**
pSorensen_tur	5.88 %	0.057	0.034	-0.010	0.124	study; driver
PCDp	1.24 %	0.012	0.012	-0.012	0.037	study
β: Species List Data (78)						
Sorensen_tur	-4.17 %	-0.043	0.026	-0.094	0.009	study
pSorensen_tur	-0.09 %	-0.001	0.010	-0.021	0.020	study
PCDp	-0.08 %	-0.001	0.006	-0.013	0.011	study
β: Resample Data (18)						
**Sorensen_tur**	**-9.6 %**	**-0.101**	**0.046**	**-0.191**	**-0.011**	**study**
**pSorensen_tur**	**-7.71 %**	**-0.080**	**0.032**	**-0.144**	**-0.017**	**study**
PCDp	-1.41 %	-0.014	0.010	-0.034	0.006	study

Table S3. Summary of LMM output for effect sizes of α and β diversity for different continents. Significant changes (i.e., 95% confidence interval does not include zero) are in bold. Abbreviations: SR, species richness; PD, Faith's phylogenetic diversity (without root); PSV: phylogenetic species variation; Sorensen_tur, Turnover component of Sorensen dissimilarity; pSorensen_tur, Turnover component of Phylogenetic Sorensen dissimilarity; PCDp, phylogenetic component of phylogenetic community dissimilarity.

index	Changes in diversity	Estimate	Std. Error	2.5 %	97.5 %	Random Terms
α: Africa (22)						
SR	-14.16 %	-0.153	0.100	-0.349	0.044	study
PD	-11.48 %	-0.122	0.082	-0.282	0.038	study
PSV	0.01 %	0.000	0.000	-0.001	0.001	study
α: Asia (27)						
**SR**	**4.38 %**	**0.043**	**0.006**	**0.031**	**0.055**	**study**
**PD**	**3.16 %**	**0.031**	**0.005**	**0.022**	**0.040**	**study**
**PSV**	**-0.12 %**	**-0.001**	**0.001**	**-0.002**	**0.000**	**study**
α: Europe (29)						
**SR**	**6.45 %**	**0.062**	**0.010**	**0.044**	**0.081**	**study**
**PD**	**3.5 %**	**0.034**	**0.005**	**0.024**	**0.044**	**study**
PSV	-0.49 %	-0.005	0.003	-0.010	0.000	study
α: North America (33)						
SR	8.68 %	0.083	0.081	-0.075	0.241	study
PD	3.17 %	0.031	0.066	-0.098	0.160	study
PSV	-1.45 %	-0.015	0.010	-0.034	0.005	study
α: Oceania (39)						
**SR**	**6.5 %**	**0.063**	**0.021**	**0.023**	**0.103**	**study**
**PD**	**4.78 %**	**0.047**	**0.010**	**0.027**	**0.067**	**study**
PSV	-2.46 %	-0.025	0.029	-0.082	0.032	study; grain_size; data_type; driver
α: South America (12)						
**SR**	**24.28 %**	**0.217**	**0.086**	**0.048**	**0.387**	**study**
**PD**	**15.78 %**	**0.147**	**0.062**	**0.025**	**0.269**	**study**
PSV	0.14 %	0.001	0.002	-0.002	0.004	study
β: Africa (22)						
Sorensen_tur	-4.96 %	-0.051	0.046	-0.142	0.040	study
pSorensen_tur	-8.09 %	-0.084	0.044	-0.170	0.002	study
PCDp	-0.92 %	-0.009	0.015	-0.039	0.021	study
β: Asia (27)						
Sorensen_tur	-9.46 %	-0.099	0.053	-0.203	0.005	study
pSorensen_tur	-5.2 %	-0.053	0.039	-0.129	0.022	study
PCDp	-0.4 %	-0.004	0.009	-0.021	0.013	study
β: Europe (29)						
Sorensen_tur	-3.47 %	-0.035	0.056	-0.146	0.075	study; grain_size
**pSorensen_tur**	**1.62 %**	**0.016**	**0.004**	**0.009**	**0.023**	**study**
PCDp	-0.05 %	0.000	0.011	-0.022	0.021	study
β: North America (33)						
**Sorensen_tur**	**-10.23 %**	**-0.108**	**0.045**	**-0.197**	**-0.019**	**study**
**pSorensen_tur**	**-6.43 %**	**-0.066**	**0.026**	**-0.118**	**-0.015**	**study**
PCDp	1.14 %	0.011	0.012	-0.012	0.035	study
β: Oceania (39)						
Sorensen_tur	-0.54 %	-0.005	0.007	-0.018	0.008	study
pSorensen_tur	2.22 %	0.022	0.046	-0.069	0.113	study
PCDp	1.11 %	0.011	0.016	-0.021	0.043	study
β: South America (12)						
Sorensen_tur	-0.38 %	-0.004	0.004	-0.012	0.004	study
pSorensen_tur	0.6 %	0.006	0.005	-0.003	0.015	study
PCDp	-2.79 %	-0.028	0.024	-0.075	0.018	study

Table S4. Changes in pairwise taxonomic β diversity when measured with Sorensen dissimilarity or its nestedness component. Both measurements gave qualitatively similar results, albeit the smaller numbers from Sorensen dissimilarity.

Groups	Sorensen	Turnover
Bird (54)	-2.73 %	-4.58 %
Fish (55)	-4.95 %	-8.89 %
Plant (53)	-4.88 %	-6.37 %
Land Use Data (66)	-4.74 %	-6.24 %
Species List Data (78)	-1.65 %	-4.17 %
Resample Data (18)	-6.37 %	-9.6 %

Table S5. Sources and characteristics of datasets collected in the study. Detailed reference information see Text S1.

Ref	location	n_site1	sp_in_phy_dat1	sp_in_phy_dat2	taxa	spatial_extent	grain_size	data_type	driver	decision
1	united_states	63	0.969	0.979	plant	region	small	old_new	ongoing disturbance	
2	canada	133	0.960	0.955	plant	region	small	old_new	ongoing disturbance	
3	united_states	3	0.962	0.938	bird	continent	moderate	land_use	urbanization	
4	india	3	0.776	0.718	bird	region	small	land_use	urbanization	excluded
5	archipelago_Antartic	4	1.000	1.000	bird	continent	high	native_exotic	invasion	
5	archipelago_Azores	9	1.000	1.000	bird	region	high	native_exotic	invasion	
5	archipelago_Canaries	11	1.000	1.000	bird	region	high	native_exotic	invasion	
5	archipelago_CapeVerdes	13	1.000	1.000	bird	region	high	native_exotic	invasion	
5	archipelago_Comoros	4	1.000	1.000	bird	region	high	native_exotic	invasion	
5	archipelago_CookIslands	15	1.000	1.000	bird	region	moderate	native_exotic	invasion	
5	archipelago_Galapagos	11	1.000	1.000	bird	region	high	native_exotic	invasion	
5	archipelago_GreaterAntilles	4	1.000	1.000	bird	region	very_high	native_exotic	invasion	
5	archipelago_Hawaii	6	1.000	1.000	bird	region	very_high	native_exotic	invasion	
5	archipelago_LesserAntilles	12	1.000	1.000	bird	region	high	native_exotic	invasion	
5	archipelago_Marianas	15	1.000	1.000	bird	region	moderate	native_exotic	invasion	
5	archipelago_NewZealand	10	1.000	1.000	bird	region	moderate	native_exotic	invasion	
5	archipelago_Pitcairns	4	1.000	1.000	bird	region	moderate	native_exotic	invasion	
5	archipelago_SocietyIslands	11	1.000	1.000	bird	region	moderate	native_exotic	invasion	
5	world_ocean	152	1.000	1.000	bird	continent	very_high	native_exotic	invasion	
5	ocean_Atlantic	45	1.000	1.000	bird	region	moderate	native_exotic	invasion	
5	ocean_Caribbean	21	1.000	1.000	bird	continent	high	native_exotic	invasion	
5	ocean_Indian	11	1.000	1.000	bird	continent	high	native_exotic	invasion	
5	ocean_Pacific	75	1.000	1.000	bird	continent	moderate	native_exotic	invasion	
6	iberian_peninsula	10	0.919	0.817	fish	region	very_high	native_exotic	invasion	
7	solomon_islands	16	0.820	0.941	plant	region	small	land_use	grazing	
7	solomon_islands	16	0.820	0.917	plant	region	small	land_use	management	
8	italy	9	0.240	0.250	plant	region	small	land_use	urbanization	excluded
9	uganda	35	0.907	0.980	bird	region	small	land_use	agriculture	
10	papua_new_guinea	6	0.934	0.940	bird	region	small	land_use	agriculture	
11	south_africa	24	0.938	0.979	bird	region	small	land_use	agriculture	
12	costa_rica	8	0.992	0.975	bird	region	small	land_use	management	
13	malaysia	20	0.929	0.964	bird	region	small	land_use	agriculture	
13	malaysia	20	0.929	0.970	bird	region	small	land_use	management	
14	sao_tome_and_principe	40	0.941	0.929	bird	region	small	land_use	agriculture	
14	sao_tome_and_principe	40	0.941	0.926	bird	region	small	land_use	management	
14	sao_tome_and_principe	40	1.000	1.000	plant	region	small	land_use	agriculture	
14	sao_tome_and_principe	40	1.000	1.000	plant	region	small	land_use	management	
15	china	15	0.864	0.835	fish	region	moderate	native_exotic	invasion	
16	australia	95	0.964	1.000	bird	region	small	land_use	grazing	
17	portugal	9	1.000	1.000	plant	region	small	land_use	management	
18	philippines	8	0.951	0.938	bird	region	small	land_use	agriculture	
19	sao_tome_and_principe	6	0.933	0.889	bird	region	small	land_use	management	
20	south_africa	56	0.990	1.000	plant	region	small	land_use	agriculture	

20	south_africa	56	0.990	1.000	plant	region	small	land_use	grazing	
20	south_africa	56	0.990	0.983	plant	region	small	land_use	management	
20	south_africa	56	0.990	0.990	plant	region	small	land_use	urbanization	
21	france	30	0.881	0.881	bird	region	moderate	land_use	urbanization	
22	costa_rica	12	0.981	1.000	bird	region	small	land_use	agriculture	
22	costa_rica	12	0.980	0.977	bird	region	small	land_use	agriculture	
23	burkina_faso	122	0.983	0.993	plant	region	small	land_use	agriculture	
24	germany	26	1.000	1.000	plant	region	small	old_new	management	
24	germany	25	0.977	0.977	plant	region	small	old_new	management	
25	united_states	14	0.825	0.773	fish	region	very_high	native_exotic	invasion	excluded
26	mexico	5	0.850	0.829	plant	region	small	land_use	management	
27	indonesia	4	0.976	0.988	plant	region	small	land_use	management	
28	costa_rica	24	0.963	1.000	bird	region	small	land_use	agriculture	
28	panama	24	0.963	1.000	bird	region	small	land_use	agriculture	
28	costa_rica	24	0.963	0.984	bird	region	small	land_use	grazing	
28	panama	24	0.963	0.984	bird	region	small	land_use	grazing	
28	costa_rica	24	0.963	0.988	bird	region	small	land_use	management	
28	panama	24	0.963	0.988	bird	region	small	land_use	management	
29	kenya	3	0.887	0.901	bird	region	small	land_use	management	
30	costa_rica	3	0.982	0.976	bird	region	small	land_use	grazing	
31	india	12	0.964	0.944	bird	region	small	land_use	management	
32	ethiopia	15	0.016	0.023	plant	region	small	land_use	management	excluded
32	ethiopia	15	0.973	1.000	plant	region	small	land_use	management	
32	ethiopia	15	0.000	0.020	plant	region	small	land_use	management	excluded
32	ethiopia	15	0.552	0.633	plant	region	small	land_use	management	excluded
33	indonesia	4	0.711	0.855	plant	region	small	land_use	management	excluded
33	indonesia	4	0.938	0.943	bird	region	small	land_use	management	
34	ethiopia	11	1.000	0.989	plant	region	small	land_use	management	
34	ethiopia	11	0.778	0.887	plant	region	small	land_use	management	excluded
34	ethiopia	11	0.762	0.722	plant	region	small	land_use	management	excluded
35	united_states	154	0.989	0.984	bird	region	small	land_use	agriculture	
35	united_states	154	0.989	0.980	bird	region	small	land_use	grazing	
35	united_states	154	0.989	0.966	bird	region	small	land_use	management	
35	united_states	154	0.989	0.983	bird	region	small	land_use	urbanization	
36	argentina	20	1.000	1.000	bird	region	small	land_use	management	
37	australia	176	1.000	1.000	plant	region	small	land_use	grazing	
38	united_kingdom	86	0.990	0.991	plant	region	small	old_new	ongoing disturbance	
39	germany	57	0.990	0.990	plant	region	high	land_use	urbanization	
39	germany	57	0.989	0.990	plant	region	high	land_use	urbanization	
40	united_states	30	1.000	0.987	plant	region	small	old_new	management	
41	china	103	0.911	0.906	fish	continent	very_high	native_exotic	invasion	
42	brazil	12	0.994	0.988	plant	region	high	old_new	post disturbance	
43	north_atlantic	35	0.885	0.806	fish	region	high	old_new	climate change	
44	united_states	43	0.820	0.810	fish	region	high	native_exotic	invasion	
44	united_states	6	0.820	0.810	fish	region	high	native_exotic	invasion	
44	united_states	7	0.784	0.797	fish	region	moderate	native_exotic	invasion	excluded
44	united_states	15	0.731	0.740	fish	region	moderate	native_exotic	invasion	excluded

44	united_states	9	0.692	0.714	fish	region	moderate	native_exotic	invasion	excluded
44	united_states	10	0.933	0.769	fish	region	moderate	native_exotic	invasion	excluded
45	canada	115	0.972	0.986	plant	region	small	old_new	ongoing disturbance	
45	canada	43	0.985	0.988	plant	region	small	old_new	ongoing disturbance	
45	canada	26	0.988	0.982	plant	region	small	old_new	ongoing disturbance	
46	romania	4	0.981	1.000	plant	region	small	land_use	grazing	
47	malaysia	6	0.904	0.903	bird	region	small	land_use	management	
48	ghana	8	0.916	0.977	bird	region	small	land_use	management	
49	kenya	48	0.928	0.924	bird	region	small	land_use	management	
50	australia	53	0.789	0.760	fish	region	very_high	native_exotic	invasion	excluded
50	australia	12	0.861	0.834	fish	continent	very_high	native_exotic	invasion	
51	spain	128	0.989	0.989	plant	region	moderate	native_exotic	invasion	
52	france	24	0.979	0.974	plant	region	small	land_use	urbanization	
53	united_states	72	0.909	0.797	fish	region	high	native_exotic	invasion	excluded
54	argentina	29	1.000	0.995	plant	region	small	old_new	grazing	
54	argentina	23	0.990	0.978	plant	region	small	old_new	grazing	
55	united_states	48	0.942	0.923	fish	continent	very_high	native_exotic	invasion	
56	eu	10	0.995	0.996	plant	continent	high	native_exotic	invasion	
56	united_kingdom	10	0.982	0.987	plant	region	high	native_exotic	invasion	
57	united_states	87	0.982	0.988	plant	region	small	old_new	ongoing disturbance	
58	united_states	60	0.982	0.961	plant	region	small	old_new	ongoing disturbance	
59	canada	48	0.895	0.917	plant	region	small	old_new	climate change	
60	comoros	20	0.875	0.913	bird	region	small	land_use	management	
61	costa_rica	42	0.830	0.918	plant	region	small	land_use	grazing	
62	comoros	15	0.941	0.917	bird	region	small	land_use	management	
63	greece	4	0.985	1.000	plant	region	small	land_use	agriculture	
64	north_america	769	0.840	0.843	bird	continent	moderate	old_new	ongoing disturbance	
65	united_states	58	0.982	0.984	plant	region	high	land_use	urbanization	
66	indonesia	63	0.805	0.856	plant	region	small	land_use	agriculture	
66	indonesia	63	0.805	0.815	plant	region	small	land_use	management	
66	indonesia	63	0.987	0.983	plant	region	small	land_use	management	
67	south_africa	10	1.000	1.000	plant	region	small	land_use	grazing	
68	spain	46	0.154	0.129	plant	region	small	land_use	grazing	excluded
68	france	46	0.154	0.129	plant	region	small	land_use	grazing	excluded
69	philippines	3	0.833	0.833	plant	region	small	land_use	agriculture	
69	philippines	3	1.000	1.000	plant	region	small	land_use	agriculture	
70	egypt	6	1.000	0.889	plant	region	small	land_use	management	
70	egypt	6	1.000	0.978	plant	region	small	land_use	urbanization	
71	argentina	48	0.989	0.994	plant	region	small	land_use	grazing	
71	chile	48	0.989	0.994	plant	region	small	land_use	grazing	
71	argentina	136	0.987	0.987	plant	region	small	land_use	grazing	
72	united_kingdom	238	0.925	0.893	plant	region	moderate	old_new	ongoing disturbance	
73	canada	8	0.791	0.756	fish	region	very_high	native_exotic	invasion	excluded
73	canada	13	0.777	0.771	fish	continent	very_high	native_exotic	invasion	excluded
74	afghanistan	3	0.941	0.889	fish	region	high	native_exotic	invasion	
74	argentina	56	0.934	0.917	fish	region	high	native_exotic	invasion	
74	australia	458	0.907	0.895	fish	continent	high	native_exotic	invasion	

74	belgium	3	0.875	0.824	fish	region	high	native_exotic	invasion	
74	brazil	92	0.963	0.961	fish	region	high	native_exotic	invasion	
74	bulgaria	4	0.915	0.897	fish	region	high	native_exotic	invasion	
74	canada	80	0.900	0.887	fish	continent	high	native_exotic	invasion	
74	chile	21	0.985	0.911	fish	region	high	native_exotic	invasion	
74	china	72	0.933	0.932	fish	continent	high	native_exotic	invasion	
74	colombia	9	0.962	0.959	fish	region	high	native_exotic	invasion	
74	congo	3	0.985	0.983	fish	region	high	native_exotic	invasion	
74	denmark	4	0.861	0.833	fish	region	high	native_exotic	invasion	
74	finland	6	0.857	0.816	fish	region	high	native_exotic	invasion	
74	france	50	0.898	0.859	fish	region	high	native_exotic	invasion	
74	french_polynesia	17	1.000	1.000	fish	region	high	native_exotic	invasion	
74	germany	4	0.887	0.831	fish	region	high	native_exotic	invasion	
74	greece	18	0.966	0.925	fish	region	high	native_exotic	invasion	
74	india	44	0.951	0.939	fish	continent	high	native_exotic	invasion	
74	indonesia	72	0.924	0.922	fish	region	high	native_exotic	invasion	
74	iran	42	0.897	0.882	fish	region	high	native_exotic	invasion	
74	ireland	4	0.857	0.840	fish	region	high	native_exotic	invasion	
74	italy	43	0.938	0.870	fish	region	high	native_exotic	invasion	
74	japan	138	0.931	0.877	fish	region	high	native_exotic	invasion	
74	kenya	8	0.972	0.942	fish	region	high	native_exotic	invasion	
74	madagascar	11	0.938	0.897	fish	region	high	native_exotic	invasion	
74	malaysia	61	0.954	0.953	fish	region	high	native_exotic	invasion	
74	martinique	5	0.929	0.895	fish	region	high	native_exotic	invasion	
74	mexico	83	0.944	0.936	fish	region	high	native_exotic	invasion	
74	morocco	12	0.923	0.875	fish	region	high	native_exotic	invasion	
74	new_caledonia	11	0.969	0.875	fish	region	high	native_exotic	invasion	
74	new_zealand	195	1.000	0.862	fish	region	high	native_exotic	invasion	
74	norway	8	0.905	0.893	fish	region	high	native_exotic	invasion	
74	panama	18	0.950	0.951	fish	region	high	native_exotic	invasion	
74	papua_new_guinea	26	0.853	0.847	fish	region	high	native_exotic	invasion	
74	philippines	6	0.892	0.867	fish	region	high	native_exotic	invasion	
74	poland	4	0.889	0.824	fish	region	high	native_exotic	invasion	
74	portugal	9	0.848	0.761	fish	region	high	native_exotic	invasion	excluded
74	russia	104	0.926	0.918	fish	continent	high	native_exotic	invasion	
74	south_africa	53	0.953	0.937	fish	region	high	native_exotic	invasion	
74	south_korea	195	0.922	0.914	fish	region	high	native_exotic	invasion	
74	spain	22	0.789	0.762	fish	region	high	native_exotic	invasion	excluded
74	sri_lanka	91	0.956	0.931	fish	region	high	native_exotic	invasion	
74	sweden	22	0.886	0.837	fish	region	high	native_exotic	invasion	
74	tanzania	13	0.979	0.978	fish	region	high	native_exotic	invasion	
74	thailand	19	0.948	0.940	fish	region	high	native_exotic	invasion	
74	tunisia	13	0.923	0.773	fish	region	high	native_exotic	invasion	excluded
74	turkey	46	0.933	0.923	fish	region	high	native_exotic	invasion	
74	united_kingdom	43	0.892	0.796	fish	region	high	native_exotic	invasion	excluded
74	united_states	193	0.959	0.944	fish	continent	high	native_exotic	invasion	
74	venezuela	17	0.943	0.941	fish	region	high	native_exotic	invasion	

74	vietnam	25	0.927	0.922	fish	region	high	native_exotic	invasion
75	ecuador	49	0.961	0.958	bird	region	small	land_use	agriculture
76	south_africa	13	0.866	0.820	bird	region	small	land_use	urbanization
77	north_america	60	0.974	0.977	plant	continent	very_high	native_exotic	invasion
77	eu	30	0.990	0.990	plant	continent	very_high	native_exotic	invasion

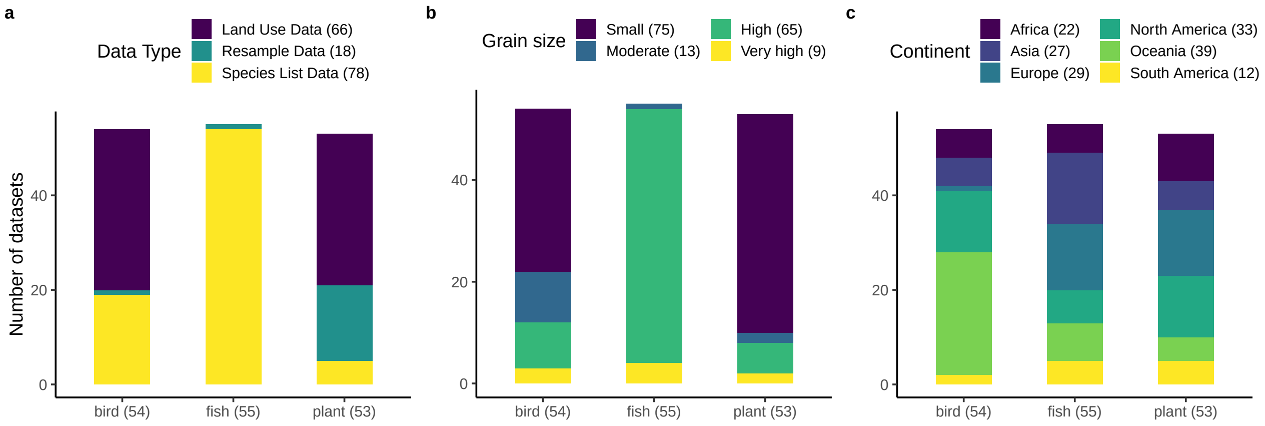

Figure S1. Sampling methods, grain sizes, and continents of datasets for the three taxonomic groups we collected. a: 54 out of 55 fish datasets were native-non-native ones; the majority of resurvey studies were about plants. b: none of the fish datasets had small grain size locales; the majority of bird and plant datasets were at small scale.

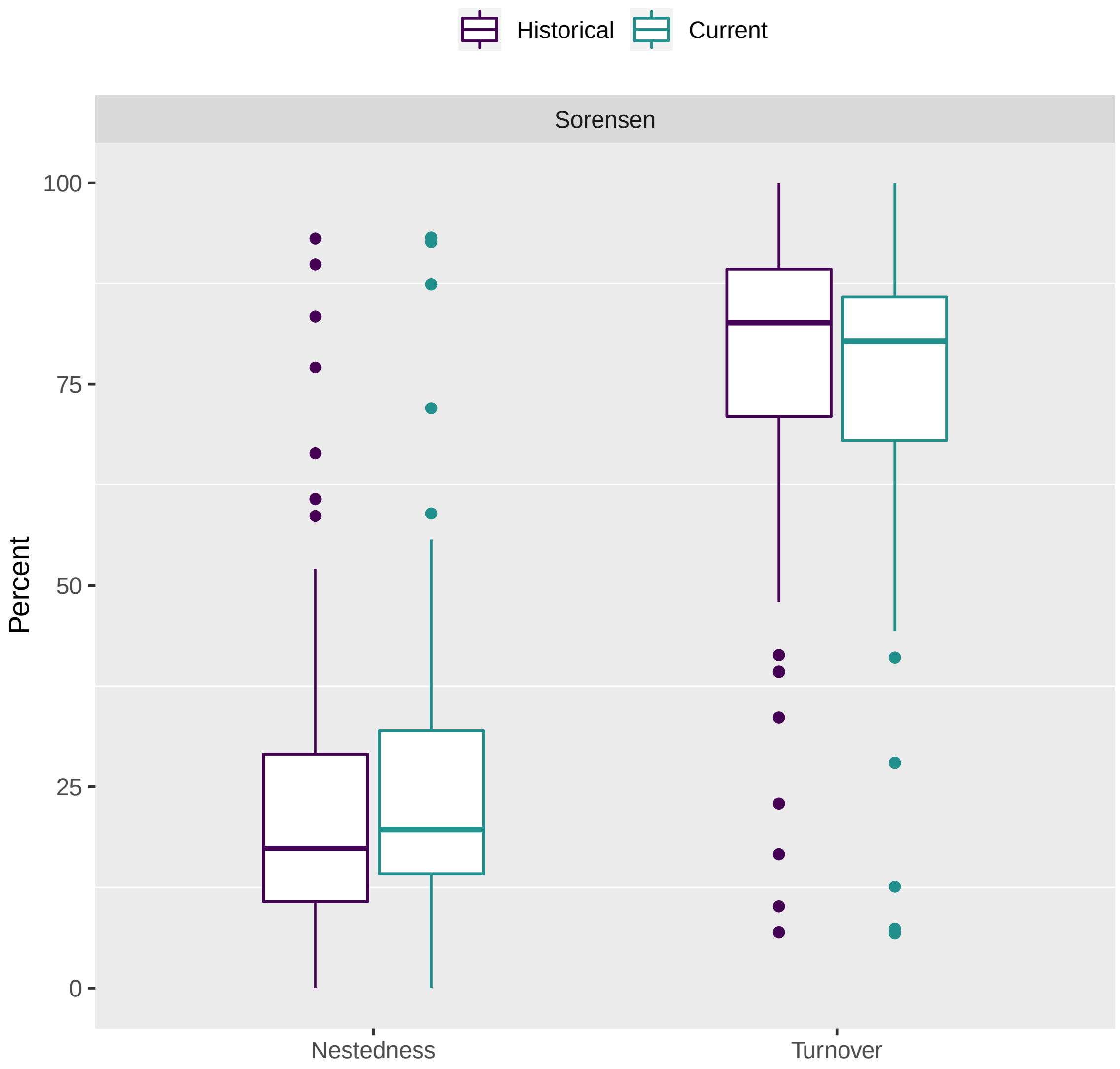

Figure S2. Partition of Sorensen dissimilarity into nestedness and turnover. Sorensen is a measure of pairwise dissimilarity of species (taxonomic) composition of assemblages.

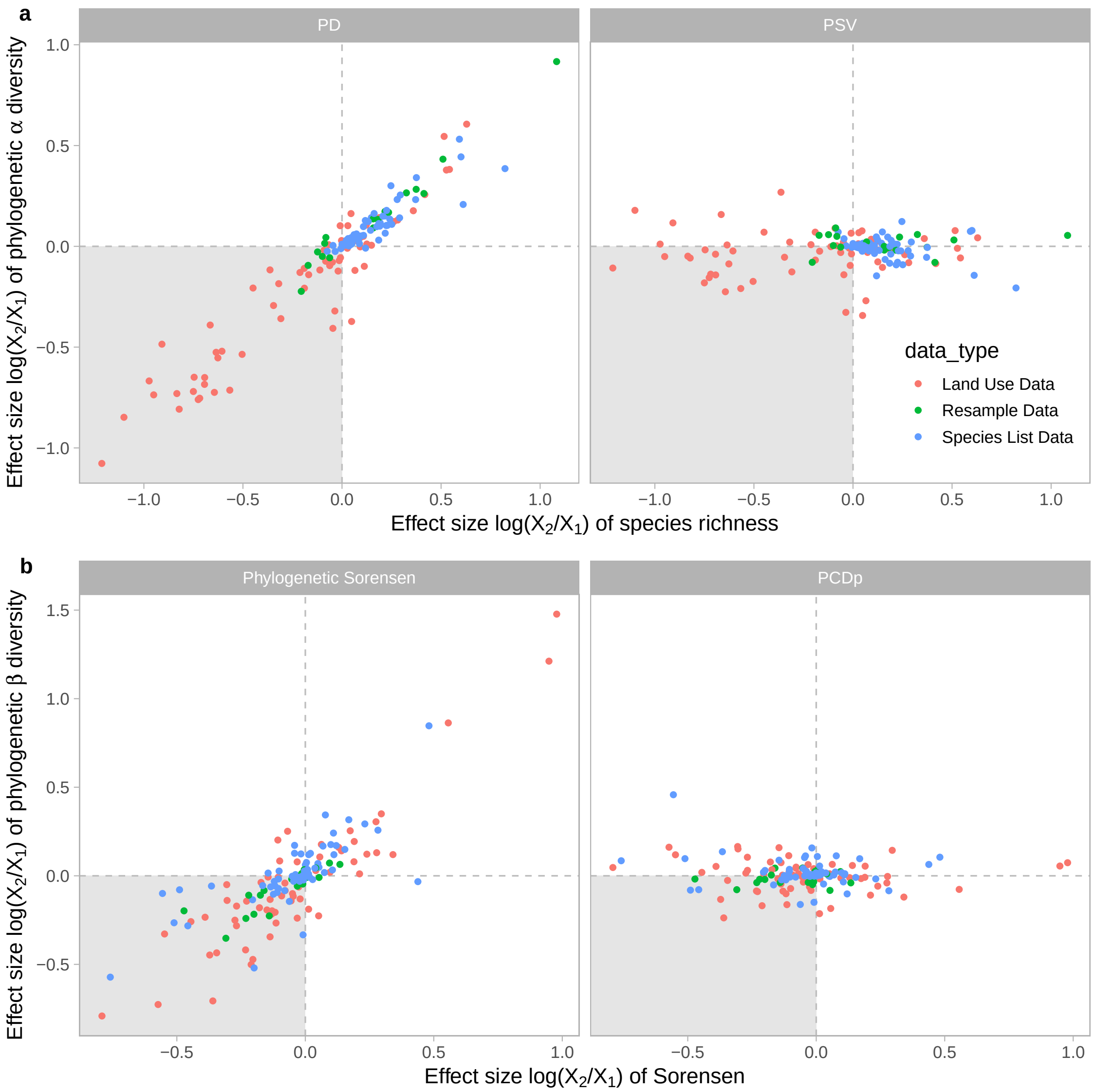

Figure S3. Changes in taxonomic diversity vs. changes in phylogenetic diversity across all datasets. A: changes in α diversity. B: changes in β diversity. Positive effect size suggests increases in site dissimilarity while negative effect size suggests decreases in site dissimilarity. Therefore, the grey area indicates both species homogenization and phylogenetic homogenization. Note that effect sizes of PSV and PCDp are independent from the effect sizes of species richness and Sorensen dissimilarity, respectively. This is not the case for `pd_unroot` (Faith's PD without root) and `psor_turnover` (turnover part of PhyloSor). Consequently, to examine patterns of "pure" phylogenetic diversity, metrices that are independent with species diversity should be used.

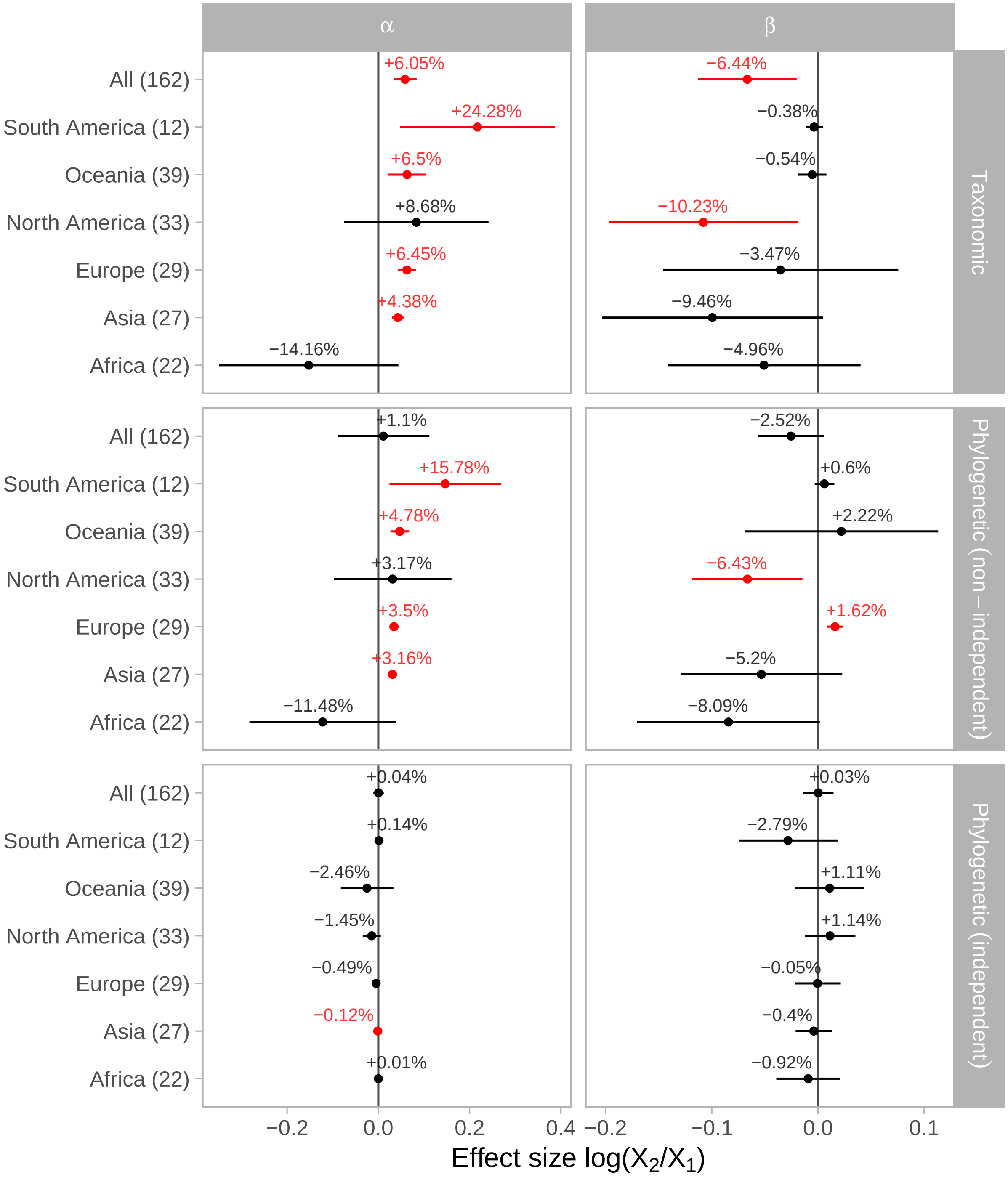

Figure S4. Changes in taxonomic and phylogenetic diversity of datasets from different continents.

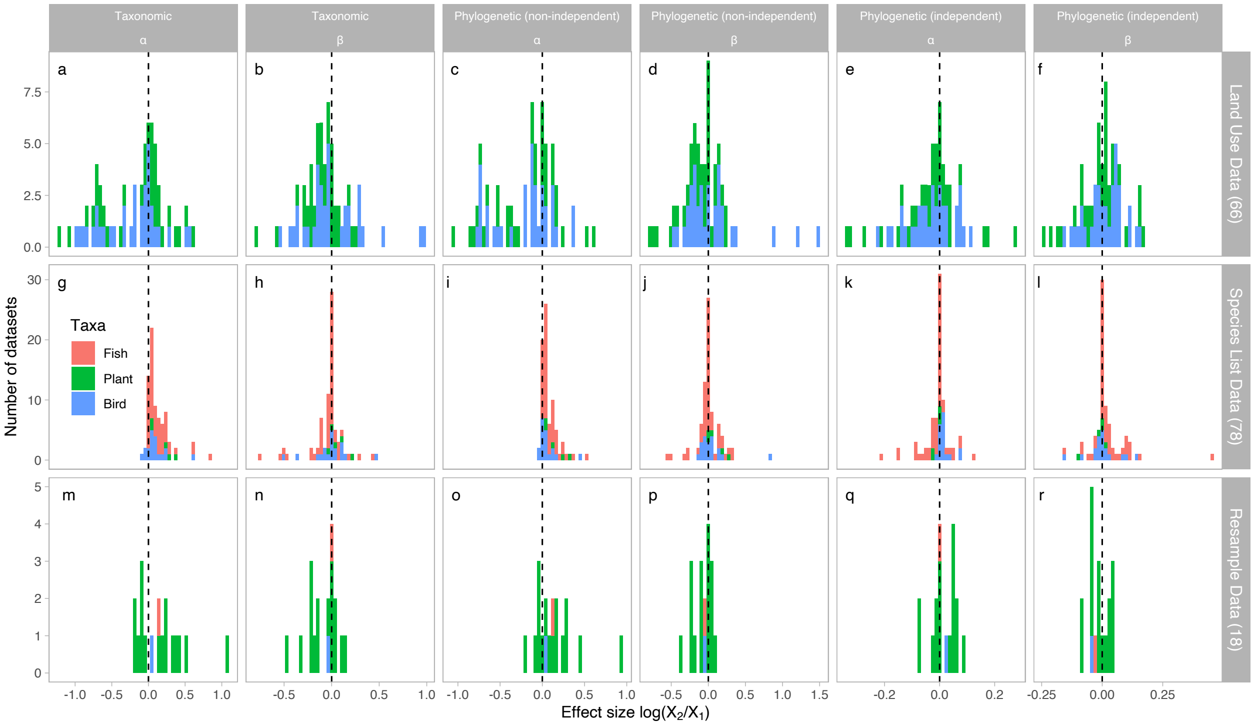

Figure S5. Histogram of log ratio effect size.

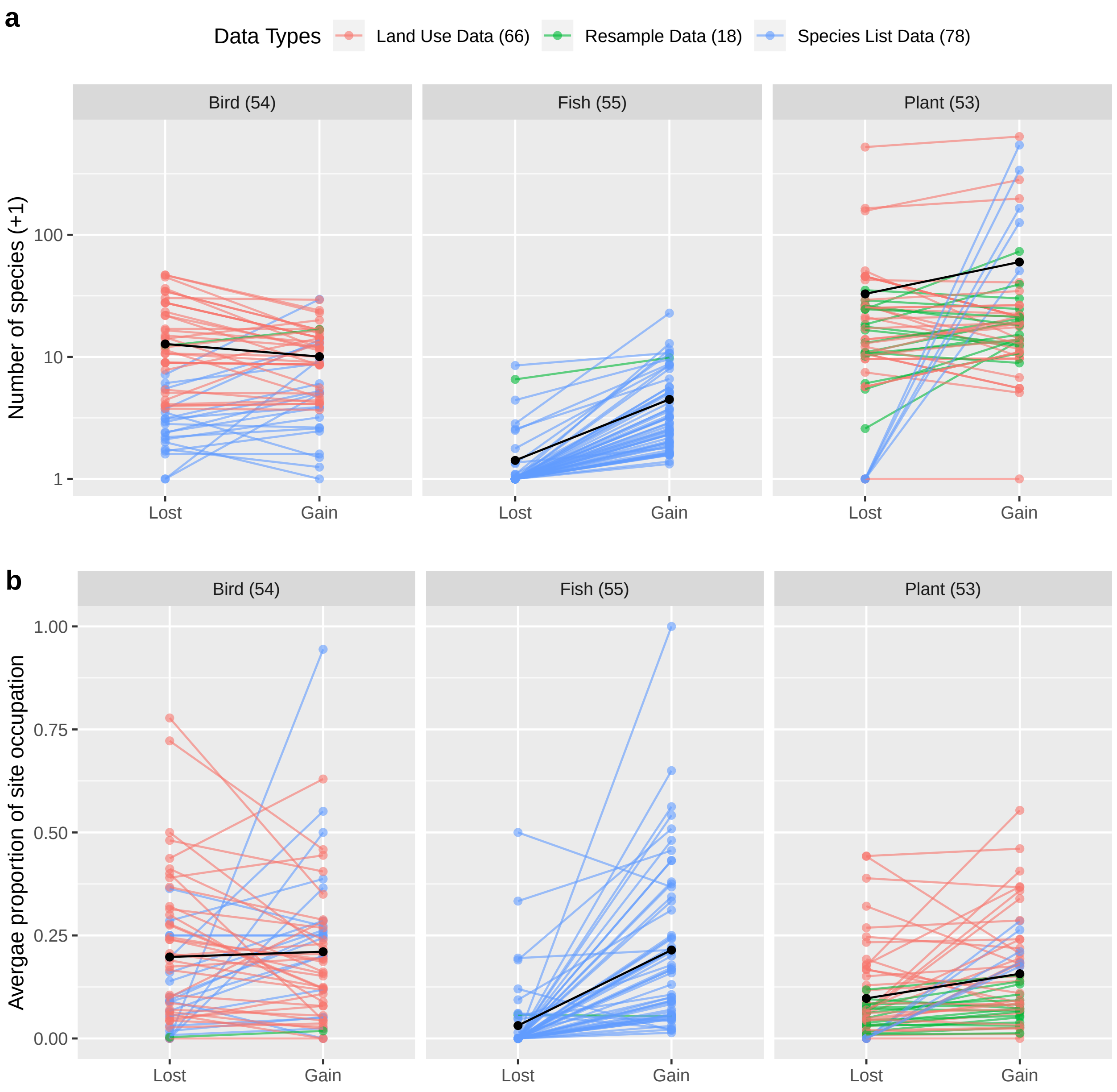

Figure S6. The average number (a) and average proportion of site occupation (b) of species that were lost and gained within datasets of different taxonomic groups. Each colored dot represents the average value of a dataset while each black dot represents mean values across all datasets. Values from the same dataset were connected with lines. Plant and fish on average have more gained species than lost ones, resulting in higher alpha diversity; gained species also occupied more sites than lost species, resulting in lower beta diversity. Note that the values in this figure were observed ones while statistics in Fig. 2 were based on weighted linear mixed models, therefore, their patterns were not exactly the same.

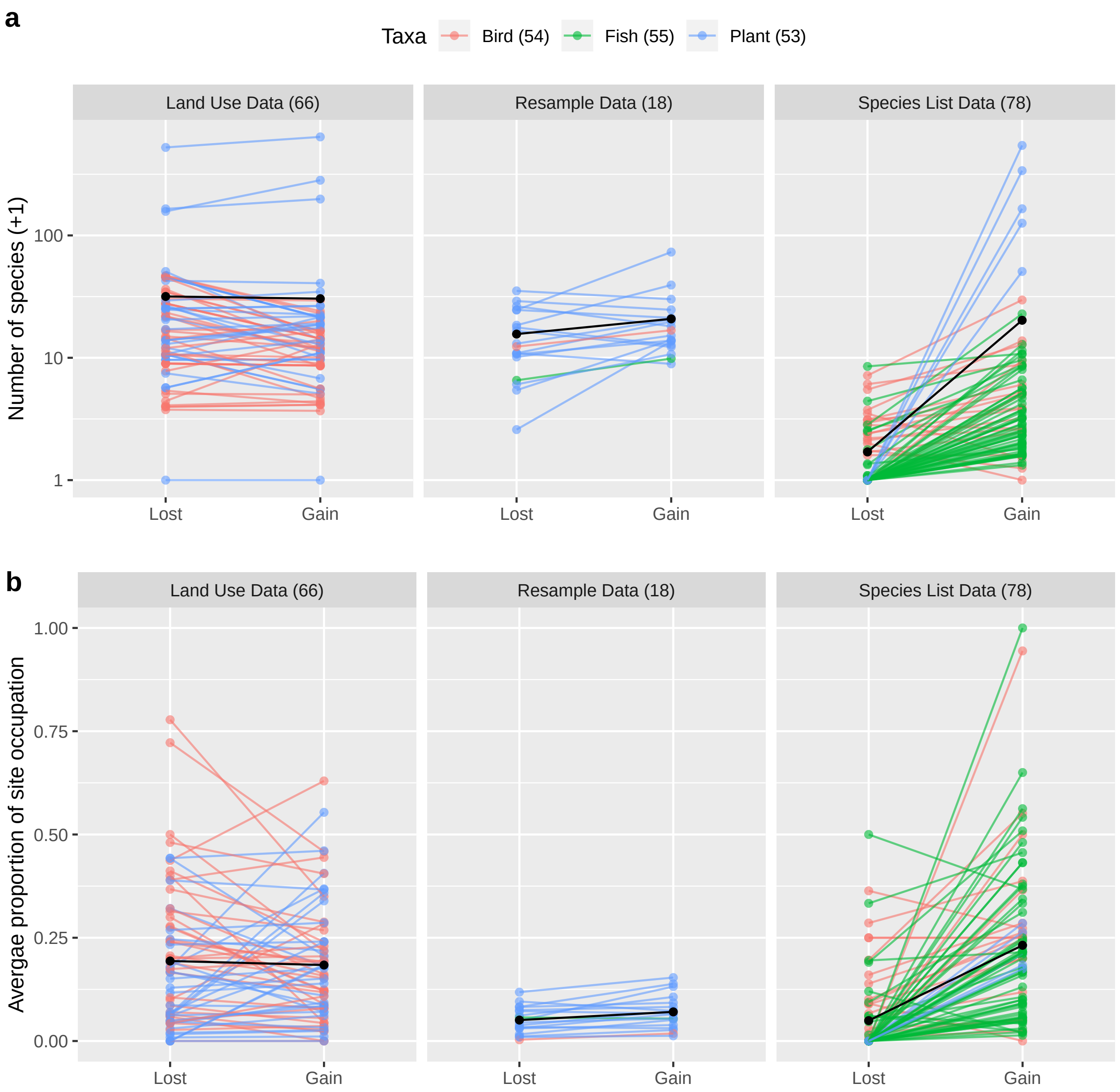

Figure S7. The average number (a) and average proportion of site occupation (b) of species that were lost and gained within datasets of different data types. Each colored dot represents the average value of a dataset while each black dot represents mean values across all datasets. Values from the same dataset were connected with lines. Datasets from `Resample Data` and `Species List Data` on average have more gained species than lost ones, resulting in higher alpha diversity; gained species also occupied more sites than lost species, resulting in lower beta diversity. Note that the values in this figure were observed ones while statistics in Fig. 3 were based on weighted linear mixed models, therefore, their patterns were not exactly the same.

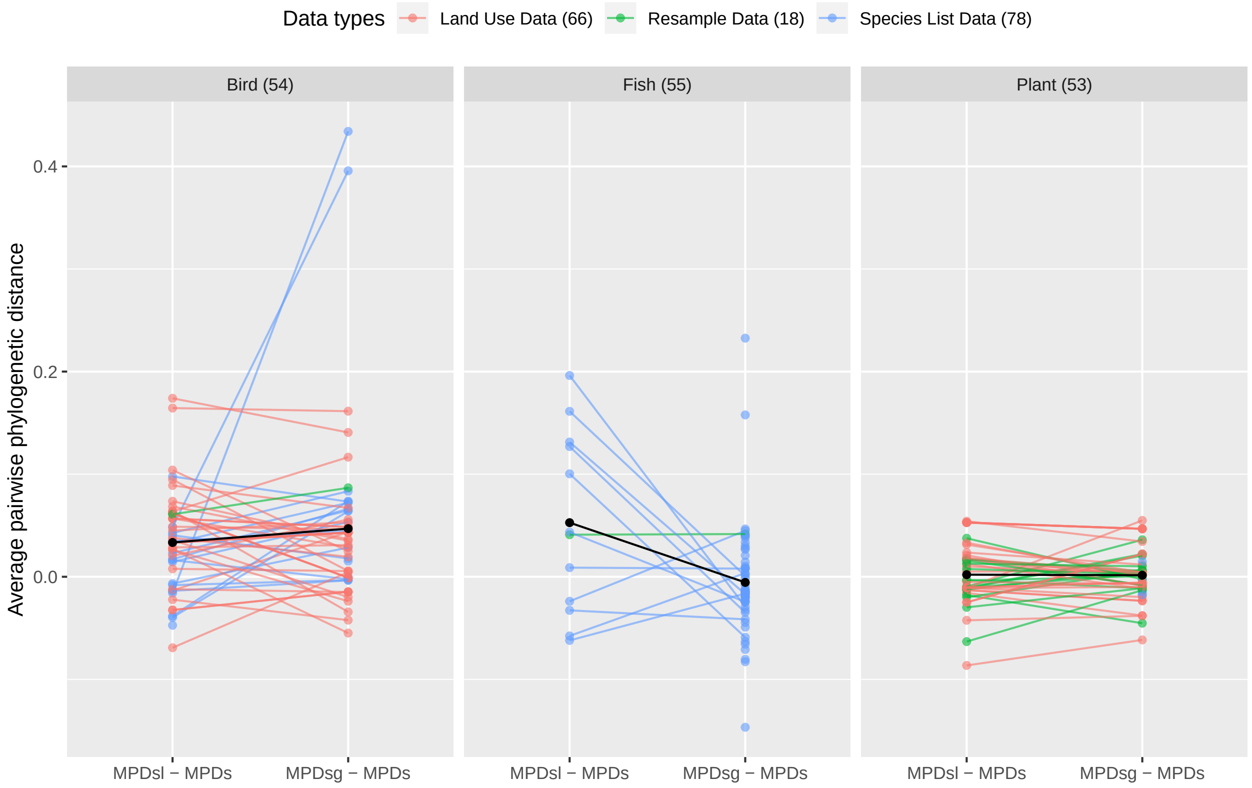

Figure S8. Site level changes in mean pairwise phylogenetic distances between gained/lost species and species that maintained of different taxonomic groups. Each colored dot represents the average value of a dataset while each black dot represents mean values across all datasets. `MPDs`: mean pairwise phylogenetic distance among shared species in both 'time periods'; `MPDsl`: mean pairwise phylogenetic distance between shared species and lost species.; `MPDsg`: mean pairwise phylogenetic distance between shared species and gained species. To account for differences of `MPDs` among datasets, we compared `MPDsl - MPDs` and `MPDsg - MPDs` for each dataset. For `Species List Data`, we set `MPDsg - MPDs` to `NA` (not available) for datasets did not have any lost species; and we only connected datasets that have lost and gained species with lines.

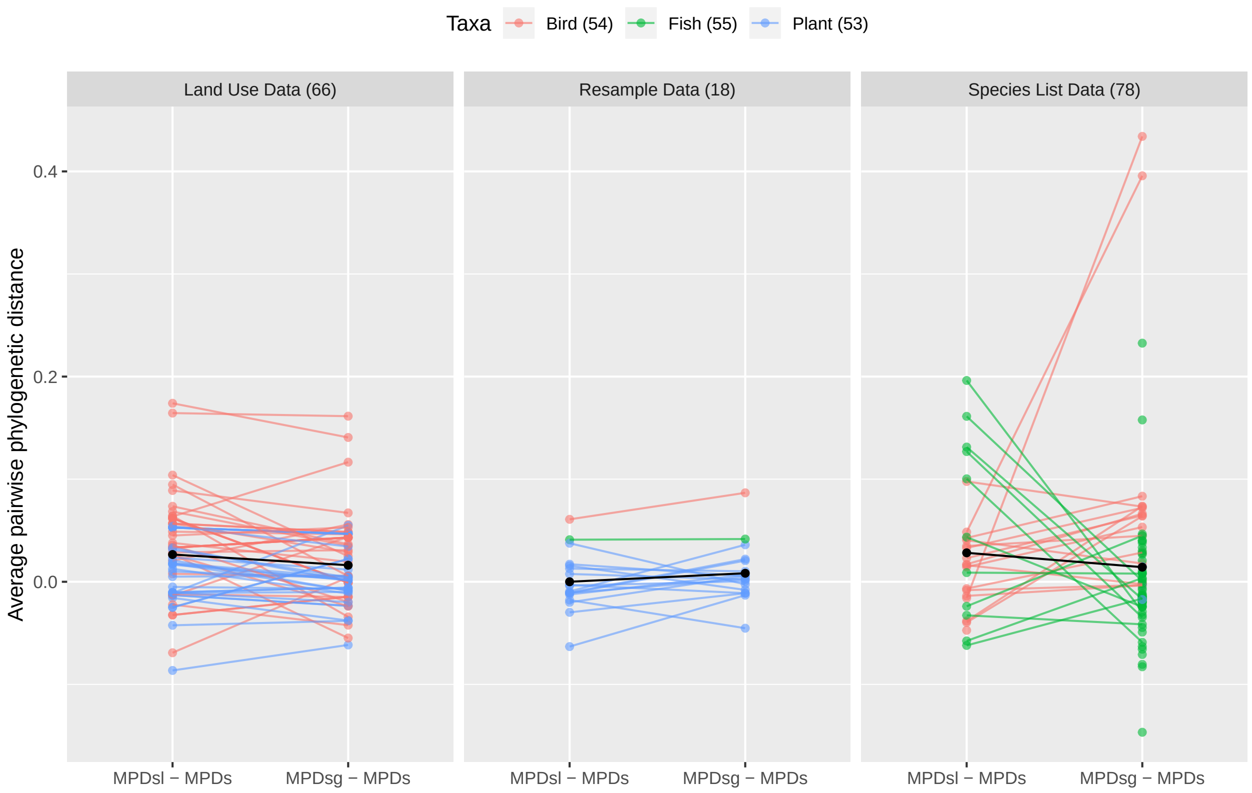

Figure S9. Site level changes in mean pairwise phylogenetic distances between gained/lost species and species that maintained of different data types. Each colored dot represents the average value of a dataset while each black dot represents mean values across all datasets. `MPDs`: mean pairwise phylogenetic distance among shared species in both 'time periods'; `MPDsl`: mean pairwise phylogenetic distance between shared species and lost species.; `MPDsg`: mean pairwise phylogenetic distance between shared species and gained species. To account for differences of `MPDs` among datasets, we compared `MPDsl - MPDs` and `MPDsg - MPDs` for each dataset. For `Species List Data`, we set `MPDsg - MPDs` to `NA` (not available) for datasets without any lost species; and we only connected datasets that have lost and gained species with lines.

Text S1: References for original studies used in this study. Numbers correspond to the "Ref" column in Supplementary Table S5.

1. Alstad, A. O. *et al.* The pace of plant community change is accelerating in remnant prairies. *Science Advances* **2**, e1500975 (2016).
2. Beauvais, M.-P., Pellerin, S. & Lavoie, C. Beta diversity declines while native plant species richness triples over 35 years in a suburban protected area. *Biological Conservation* **195**, 73–81 (2016).
3. Blair, R. B. & Johnson, E. M. Suburban habitats and their role for birds in the urban–rural habitat network: Points of local invasion and extinction? *Landscape Ecology* **23**, 1157–1169 (2008).
4. Naithani, A. & Bhatt, D. Bird community structure in natural and urbanized habitats along an altitudinal gradient in pauri district (garhwal himalaya) of uttarakhand state, india. *Biologia* **67**, 800–808 (2012).
5. Cassey, P., Lockwood, J. L., Blackburn, T. M. & Olden, J. D. Spatial scale and evolutionary history determine the degree of taxonomic homogenization across island bird assemblages. *Diversity and Distributions* **13**, 458–466 (2007).
6. Clavero, M. & Garcı'a-Berthou, E. Homogenization dynamics and introduction routes of invasive freshwater fish in the iberian peninsula. *Ecological Applications* **16**, 2313–2324 (2006).
7. Katovai, E., Burley, A. L. & Mayfield, M. M. Understory plant species and functional diversity in the degraded wet tropical forests of kolombangara island, solomon islands. *Biological Conservation* **145**, 214–224 (2012).
8. Giordano, S. *et al.* Biodiversity and trace element content of epiphytic bryophytes in urban and extraurban sites of southern italy. *Plant Ecology* **170**, 1–14 (2004).
9. Naidoo, R. Species richness and community composition of songbirds in a tropical forest-agricultural landscape. in *Animal conservation forum* **7**, 93–105 (Cambridge University Press, 2004).
10. Dawson, J. *et al.* Bird communities of the lower waria valley, morobe province, papua new guinea: A comparison between habitat types. *Tropical Conservation Science* **4**, 317–348 (2011).
11. Neuschulz, E. L., Botzat, A. & Farwig, N. Effects of forest modification on bird community composition and seed removal in a heterogeneous landscape in south africa. *Oikos* **120**, 1371–1379 (2011).
12. Reid, J. L., Harris, J. B. C. & Zahawi, R. A. Avian habitat preference in tropical forest restoration in southern costa rica. *Biotropica* **44**, 350–359 (2012).
13. Azhar, B. *et al.* The influence of agricultural system, stand structural complexity and landscape context on foraging birds in oil palm landscapes. *Ibis* **155**, 297–312 (2013).

14. Lima, R. F. de, Dallimer, M., Atkinson, P. W. & Barlow, J. Biodiversity and land use change: Understanding the complex responses of an endemic-rich bird assemblage. *Diversity and Distributions* **19**, 411–422 (2013).

15. Ding, C., Jiang, X., Xie, Z. & Brosse, S. Seventy-five years of biodiversity decline of fish assemblages in chinese isolated plateau lakes: Widespread introductions and extirpations of narrow endemics lead to regional loss of dissimilarity. *Diversity and Distributions* **23**, 171–184 (2017).

16. Woinarski, J. *et al.* Fauna assemblages in regrowth vegetation in tropical open forests of the northern territory, australia. *Wildlife Research* **36**, 675–690 (2010).

17. Proença, V. M., Pereira, H. M., Guilherme, J. & Vicente, L. Plant and bird diversity in natural forests and in native and exotic plantations in nw portugal. *Acta Oecologica* **36**, 219–226 (2010).

18. Mallari, N. *et al.* Population densities of understorey birds across a habitat gradient in palawan, philippines: Implications for conservation. *Oryx* **45**, 234–242 (2011).

19. Dallimer, M., Parnell, M., Bicknell, J. E. & Melo, M. The importance of novel and agricultural habitats for the avifauna of an oceanic island. *Journal for Nature Conservation* **20**, 191–199 (2012).

20. Siebert, S. J. Patterns of plant species richness of temperate and tropical grassland in south africa. *Plant Ecology and Evolution* **144**, 249–254 (2011).

21. Filippi-Codaccioni, O., Devictor, V., Clobert, J. & Julliard, R. Effects of age and intensity of urbanization on farmland bird communities. *Biological Conservation* **141**, 2698–2707 (2008).

22. Frishkoff, L. O. *et al.* Loss of avian phylogenetic diversity in neotropical agricultural systems. *Science* **345**, 1343–1346 (2014).

23. Devineau, J.-L., Fournier, A. & Nignan, S. 'Ordinary biodiversity' in western burkina faso (west africa): What vegetation do the state forests conserve? *Biodiversity and Conservation* **18**, 2075 (2009).

24. Heinrichs, S. & Schmidt, W. Biotic homogenization of herb layer composition between two contrasting beech forest communities on limestone over 50 years. *Applied Vegetation Science* **20**, 271–281 (2017).

25. Hoagstrom, C. W., Wall, S. S., Kral, J. G., Blackwell, B. G. & Berry Jr, C. R. Zoogeographic patterns and faunal change of south dakota fishes. *Western North American Naturalist* **67**, 161–184 (2007).

26. Hietz, P. Conservation of vascular epiphyte diversity in mexican coffee plantations. *Conservation Biology* **19**, 391–399 (2005).

27. Kessler, M. *et al.* Tree diversity in primary forest and different land use systems in central sulawesi, indonesia. *Biodiversity & Conservation* **14**, 547–560 (2005).

28. Ranganathan, J., Chan, K. & Daily, G. C. Satellite detection of bird communities in tropical

countryside. *Ecological Applications* **17**, 1499–1510 (2007).

29. Farwig, N., Sajita, N. & Böhning-Gaese, K. Conservation value of forest plantations for bird communities in western kenya. *Forest Ecology and Management* **255**, 3885–3892 (2008).
30. Gomes, L. G., Oostra, V., Nijman, V., Cleef, A. M. & Kappelle, M. Tolerance of frugivorous birds to habitat disturbance in a tropical cloud forest. *Biological Conservation* **141**, 860–871 (2008).
31. Ranganathan, J., Daniels, R. R., Chandran, M. S., Ehrlich, P. R. & Daily, G. C. Sustaining biodiversity in ancient tropical countryside. *Proceedings of the National Academy of Sciences* **105**, 17852–17854 (2008).
32. Hylander, K. & Nemomissa, S. Complementary roles of home gardens and exotic tree plantations as alternative habitats for plants of the ethiopian montane rainforest. *Conservation Biology* **23**, 400–409 (2009).
33. Kessler, M. *et al.* Alpha and beta diversity of plants and animals along a tropical land use gradient. *Ecological Applications* **19**, 2142–2156 (2009).
34. Schmitt, C. B., Senbeta, F., Denich, M., Preisinger, H. & Boehmer, H. J. Wild coffee management and plant diversity in the montane rainforest of southwestern ethiopia. *African Journal of Ecology* **48**, 78–86 (2010).
35. Chapman, K. A. & Reich, P. B. Land use and habitat gradients determine bird community diversity and abundance in suburban, rural and reserve landscapes of minnesota, usa. *Biological Conservation* **135**, 527–541 (2007).
36. Lantschner, M. V., Rusch, V. & Peyrou, C. Bird assemblages in pine plantations replacing native ecosystems in nw patagonia. *Biodiversity and Conservation* **17**, 969–989 (2008).
37. Fensham, R., Dwyer, J., Eyre, T., Fairfax, R. & Wang, J. The effect of clearing on plant composition in mulga (acacia aneura) dry forest, australia. *Austral Ecology* **37**, 183–192 (2012).
38. Keith, S. A., Newton, A. C., Morecroft, M. D., Bealey, C. E. & Bullock, J. M. Taxonomic homogenization of woodland plant communities over 70 years. *Proceedings of the Royal Society of London B: Biological Sciences* **276**, 3539–3544 (2009).
39. Kühn, I. & Klotz, S. Urbanization and homogenization–comparing the floras of urban and rural areas in germany. *Biological Conservation* **127**, 292–300 (2006).
40. Li, D. & Waller, D. Drivers of observed biotic homogenization in pine barrens of central wisconsin. *Ecology* **96**, 1030–1041 (2015).
41. Liu, C., He, D., Chen, Y. & Olden, J. D. Species invasions threaten the antiquity of china's freshwater fish fauna. *Diversity and Distributions* **23**, 556–566 (2017).
42. Lôbo, D., Leão, T., Melo, F. P., Santos, A. M. & Tabarelli, M. Forest fragmentation drives atlantic forest

of northeastern brazil to biotic homogenization. *Diversity and Distributions* **17**, 287–296 (2011).

43. Magurran, A. E., Dornelas, M., Moyes, F., Gotelli, N. J. & McGill, B. Rapid biotic homogenization of marine fish assemblages. *Nature Communications* **6**, 8405 (2015).
44. Marchetti, M. P., Lockwood, J. L. & Light, T. Effects of urbanization on california's fish diversity: Differentiation, homogenization and the influence of spatial scale. *Biological Conservation* **127**, 310–318 (2006).
45. McCune, J. L. & Vellend, M. Gains in native species promote biotic homogenization over four decades in a human-dominated landscape. *Journal of Ecology* **101**, 1542–1551 (2013).
46. Baur, B. *et al.* Effects of abandonment of subalpine hay meadows on plant and invertebrate diversity in transylvania, romania. *Biological Conservation* **132**, 261–273 (2006).
47. Sheldon, F. H., Styring, A. & Hosner, P. A. Bird species richness in a bornean exotic tree plantation: A long-term perspective. *Biological Conservation* **143**, 399–407 (2010).
48. Phalan, B., Onial, M., Balmford, A. & Green, R. E. Reconciling food production and biodiversity conservation: Land sharing and land sparing compared. *Science* **333**, 1289–1291 (2011).
49. Munyekenye, F., Mwangi, E. & Gichuki, N. Bird species richness and abundance in different forest types at kakamega forest, western kenya. *Ostrich-Journal of African Ornithology* **79**, 37–42 (2008).
50. Olden, J. D., Kennard, M. J. & Pusey, B. J. Species invasions and the changing biogeography of australian freshwater fishes. *Global Ecology and Biogeography* **17**, 25–37 (2008).
51. Pino, J., Font, X., De Cáceres, M. & Molowny-Horas, R. Floristic homogenization by native ruderal and alien plants in north-east spain: The effect of environmental differences on a regional scale. *Global Ecology and Biogeography* **18**, 563–574 (2009).
52. Plue, J. *et al.* Persistent changes in forest vegetation and seed bank 1,600 years after human occupation. *Landscape Ecology* **23**, 673–688 (2008).
53. Pool, T. K. & Olden, J. D. Taxonomic and functional homogenization of an endemic desert fish fauna. *Diversity and Distributions* **18**, 366–376 (2012).
54. Puhl, L., Perelman, S., Batista, W., Burkart, S. & León, R. Local and regional long-term diversity changes and biotic homogenization in two temperate grasslands. *Journal of Vegetation Science* **25**, 1278–1288 (2014).
55. Rahel, F. J. Homogenization of fish faunas across the united states. *Science* **288**, 854–856 (2000).
56. Ricotta, C. *et al.* Phylogenetic beta diversity of native and alien species in european urban floras. *Global Ecology and Biogeography* **21**, 751–759 (2012).
57. Rogers, D. A., Rooney, T. P., Olson, D. & Waller, D. M. Shifts in southern wisconsin forest canopy and

understory richness, composition, and heterogeneity. *Ecology* **89**, 2482–2492 (2008).

58. Rooney, T. P., Wiegmann, S. M., Rogers, D. A. & Waller, D. M. Biotic impoverishment and homogenization in unfragmented forest understory communities. *Conservation Biology* **18**, 787–798 (2004).
59. Savage, J. & Vellend, M. Elevational shifts, biotic homogenization and time lags in vegetation change during 40 years of climate warming. *Ecography* **38**, 546–555 (2015).
60. Doulton, H., Marsh, C., Newman, A., Bird, K. & Bell, M. Conservation comoros 2005: Biodiversity and resource-use assessment and environmental awareness, final report. *Unpublished report* (2007).
61. Mayfield, M. M., Ackerly, D. & Daily, G. C. The diversity and conservation of plant reproductive and dispersal functional traits in human-dominated tropical landscapes. *Journal of Ecology* **94**, 522–536 (2006).
62. Marsh, C. J., Lewis, O. T., Said, I. & Ewers, R. M. Community-level diversity modelling of birds and butterflies on anjouan, comoro islands. *Biological Conservation* **143**, 1364–1374 (2010).
63. Kati, V., Zografou, K., Tzirkalli, E., Chitos, T. & Willemse, L. Butterfly and grasshopper diversity patterns in humid mediterranean grasslands: The roles of disturbance and environmental factors. *Journal of Insect Conservation* **16**, 807–818 (2012).
64. Schipper, A. M. *et al.* Contrasting changes in the abundance and diversity of north american bird assemblages from 1971 to 2010. *Global Change Biology* **22**, 3948–3959 (2016).
65. Schwartz, M. W., Thorne, J. H. & Viers, J. H. Biotic homogenization of the california flora in urban and urbanizing regions. *Biological Conservation* **127**, 282–291 (2006).
66. Sheil, D. *et al. Exploring biological diversity, environment, and local people's perspectives in forest landscapes: Methods for a multidisciplinary landscape assessment.* (CIFOR, 2002).
67. O'Connor, T. Influence of land use on plant community composition and diversity in highland sourveld grassland in the southern drakensberg, south africa. *Journal of Applied Ecology* **42**, 975–988 (2005).
68. Medina, R. *et al.* Epiphytic bryophytes in harsh environments: The juniperus thurifera forests. *Journal of Bryology* **32**, 23–31 (2010).
69. Center for International Forestry Research (CIFOR). Multidisciplinary Landscape Assessment - Philippines. (2013).
70. Norfolk, O., Eichhorn, M. P. & Gilbert, F. Traditional agricultural gardens conserve wild plants and functional richness in arid south sinai. *Basic and Applied Ecology* **14**, 659–669 (2013).
71. Peri, P. L., Lencinas, M. V., Martı'nez Pastur, G., Wardell-Johnson, G. W. & Lasagno, R. Diversity

patterns in the steppe of argentinean southern patagonia: Environmental drivers and impact of grazing. *Steppe ecosystems: Biological diversity, Management and Restoration* 346 (2013).

72. Smart, S. M. *et al.* Biotic homogenization and changes in species diversity across human-modified ecosystems. *Proceedings of the Royal Society of London B: Biological Sciences* **273**, 2659–2665 (2006).
73. Taylor, E. B. An analysis of homogenization and differentiation of canadian freshwater fish faunas with an emphasis on british columbia. *Canadian Journal of Fisheries and Aquatic Sciences* **61**, 68–79 (2004).
74. Tedesco, P. A. *et al.* A global database on freshwater fish species occurrence in drainage basins. *Scientific Data* **4**, (2017).
75. O'Dea, N. & Whittaker, R. J. How resilient are andean montane forest bird communities to habitat degradation? *Biodiversity and Conservation* **16**, 1131–1159 (2007).
76. Rensburg, B. J. van, Peacock, D. S. & Robertson, M. P. Biotic homogenization and alien bird species along an urban gradient in south africa. *Landscape and Urban Planning* **92**, 233–241 (2009).
77. Winter, M. *et al.* The role of non-native plants and vertebrates in defining patterns of compositional dissimilarity within and across continents. *Global Ecology and Biogeography* **19**, 332–342 (2010).
